# *Myrcia bella* Leaf Extract Presents Hypoglycemic Activity via PI3k/Akt Insulin Signaling Pathway

**DOI:** 10.1155/2014/543606

**Published:** 2014-04-27

**Authors:** Priscilla Maria Ponce Vareda, Luiz Leonardo Saldanha, Nathalia Aparecida de Paula Camaforte, Natalia Moretti Violato, Anne Lígia Dokkedal, José Roberto Bosqueiro

**Affiliations:** ^1^Institute of Biosciences, São Paulo State University (UNESP), 18618-970 Botucatu, SP, Brazil; ^2^Department of Biological Sciences, Faculty of Sciences, São Paulo State University (UNESP), 17033-360 Bauru, SP, Brazil; ^3^Department of Physical Education, Faculty of Sciences, São Paulo State University (UNESP), Avenida Engenheiro Luiz Edmundo Carrijo Coube, 14-01, 17033-360 Bauru, SP, Brazil

## Abstract

Species of *Myrcia* are used by indigenous people and in traditional communities in Brazil for the treatment of *Diabetes mellitus*. We investigated the hypoglycemic effect of the extract of leaves of *Myrcia bella* in diabetic mice. The chemical fingerprinting of the 70% EtOH extract characterized as main constituents flavonoid aglycones, flavonoid-*O*-glycosides, and acylated flavonoid-*O*-glycosides derivatives of quercetin and myricetin. Mice were treated with saline or extract of *M. bella* (300 or 600 mg/Kg b.w.) for 14 days. Body weight and water and food intake were measured every day. Fasting blood glucose was measured weekly. At the end of the treatment, blood insulin, triglycerides, total cholesterol, and protein were measured. Glycogen content and expression of proteins of the insulin signaling pathway were measured in liver. The treatment with 600 mg/Kg reduced the fasting blood glucose in diabetic mice of the 7th day as water and food intake and increased hepatic glycogen. Total cholesterol and triglycerides were reduced in diabetic treated mice. The treatment increased the expression of IRS-1, PI3-K, and AKT in the livers of diabetic treated mice. The results indicate that the extract of the leaves of *Myrcia bella* has hypoglycemic properties and possibly acts to regulate glucose uptake by the liver.

## 1. Introduction

Diabetes is a metabolic disorder characterized by chronic hyperglycemia as a result of defects in insulin action, insulin secretion, or both. This disorder results in abnormality in protein, lipid, and carbohydrate metabolism, leading to long-term complications, such as neuropathy, nephropathy, retinopathy, atherosclerosis, and peripheral vascular disease [1]. According to the report of world health organization (WHO), the world is now in the middle of a diabetes epidemic. In 1985, it was estimated that there were 30 million people with diabetes worldwide, and this number dramatically increased in the ensuing 10 years, reaching 135 million people. Estimates indicate that this number, by 2030, should grow to 366 million people with this syndrome, of which 90% will develop type 2 diabetes (DM2). Today, it is estimated that there are about 12 million diabetic patients in Brazil [2].

Current treatments for diabetes include the use of exogenous insulin and the administration of allopathic drugs, such as sulfonylureas (glibenclamide), biguanides (metformin), and alpha-glucosidase inhibitors (acarbose and miglitol). The oral hypoglycemic agents exert their effects in reducing glycemia through a variety of mechanisms that include reducing hepatic glucose production, increasing insulin secretion by pancreatic beta cells, improving insulin sensitivity, and inhibiting digestion and intestinal absorption of glucose [3]. However, these drugs may cause side effects, decreasing response after prolonged use, and there is also a high financial cost to the patient [4]. Based on this information, research for new antidiabetic agents that maintain the same therapeutic efficacy with fewer side effects is necessary [5].

*Myrcia bella* is a plant belonging to the Myrtaceae family. This family consists of 133 genera and more than 3,800 species with two main centers of development in tropical America and Australia, although they occur in other regions of the world [6].* Myrcia* is one of the largest genera in the Myrtaceae family with about 760 species and is well represented throughout the Brazilian territory [7]. Species of* Myrcia* have been used by indigenous and in traditional communities in Brazil as astringents and diuretics and for the treatment of hypertension and* Diabetes mellitus* [8, 9].

The plants known as “pedra-hume-caá” or insulin plants have been used for the treatment of diabetes according to the empirical African and indigenous knowledge that influenced the formation of the Brazilian culture [10]. The species related to “pedra-hume-caá” are* Myrcia sphaerocarpa, Myrcia citrifolia, Myrcia guianensis, Myrcia uniflora, Myrcia multiflora, Myrcia salicifolia, and Myrcia speciosa* [11–14]. Despite the large number of species within the genus, there are few studies that assess their potential hypoglycemic properties, and there is no experimental evidence that proves the hypoglycemic effect of* Myrcia bella*.

The aim of the present study was to evaluate the hypoglycemic effect of the extract of the leaves of* Myrcia bella* through the investigation of metabolic, biochemical, and molecular parameters. Body weight, food and water intake, fasting glycemia, blood triglycerides, total cholesterol and protein, basal plasma insulin, hepatic and muscular glycogen, and the expression of some proteins of the insulin signaling pathway in the liver of control and streptozotocin-diabetic mice were investigated.

## 2. Material and Methods

### 2.1. Plant Material and Extraction

The leaves of* M. bella *were collected in November 2010 at the Jardim Botânico Municipal de Bauru, SP, Brazil (22°20′30′′ S and 49°00′30′′ W). Voucher specimens were prepared, identified, and deposited at the Herbarium of the UNESP—Univ Estadual Paulista “Júlio de Mesquita Filho”—UNBA (Bauru, SP, Brazil) under number 5508. Fresh leaves were dried at 40°C for 48 h. The separated powdered leaves (1.3 kg) were extracted with EtOH/H_2_O (7 : 3* v*/*v*) by percolation at room temperature. The filtrate was concentrated to dryness under reduced pressure at 40°C furnishing the hydroalcoholic extract (364 g).

### 2.2. Chemical Profiling and Identification

The chemical composition of the crude extract of* M. bella* was investigated by mass spectrometry using an Accela (Thermo Scientific, San Jose, CA, USA) LCQ Fleet with Ion Trap 3D and ionization by eletrospray (ESI) in negative ion mode. The 70% EtOH extract was dissolved in MeOH–H_2_O (8 : 2) and diluted at final concentration of 1 *μ*g mL^−1^. This solution was infused in the ESI source by flow injection analysis (FIA) using a syringe pump; the flow rate was 33 *μ*L·min^−1^. The capillary voltage was −20 V, the spray voltage was 4 kV, and the tube lens offset was −55 V. The capillary temperature was 275°C. Nitrogen was used both as drying gas at a flow rate of 60 (arbitrary units) and as nebulising gas. The nebulizer temperature was set at 280°C, and a potential of −4 V was used on the capillary. Negative ion mass spectra were recorded in the range* m/z* 100–1050. Two scan events were prescribed to run in the LCQ mass spectrometry. The first event was a full-scan spectrum to acquire data on the deprotonated compounds within the scan range. The second scan event was a MS/MS^*n*^ experiment performed using a data dependent scan on deprotonated molecule [M–H]^−^. The collision energy for MS/MS^*n*^ was adjusted to 10–25%. The constituents present in the 70% EtOH extract were identified by comparison of their MS/MS^*n*^ data with data from the literature [15].

### 2.3. Animals

Male Swiss mice (aged 2 months, weighting 20–30 g) were obtained from the Institute of Biosciences, São Paulo State University, Botucatu, SP, Brazil. The animals were kept under standard environmental conditions (22 ± 2°C) and 12 h light/dark cycle. Water and food were given* at libitum*. The animals were killed by CO_2_ exposure, followed by decapitation. The experimental protocol was approved by the Animal Experimentation Ethics Committee of UNESP/Araçatuba (Process no. 01741-2012).

### 2.4. Acute Toxicity Test

Male Swiss normal mice were divided into 2 groups of 10 animals (CTL SAL and CTL EXT) and fasted during 4 hours. After this period saline (1 mL/Kg) or the extract of* Myrcia bella* (2,000 mg/Kg) was administered by gavage. The animals had their behavior analyzed at 30, 60, 90, 120, 240, and 360 minutes after the gavage according to the Hippocratic screening described by Brito [16]. The mice were observed and weighted during 14 days. At the end of the observation period, the animals were killed and liver, kidneys, lungs, heart, and spleen were collected and weighted. This screening provides a general estimate of the toxicity of the substance of the conscious state and general disposal, activity, and coordination of the motor system, reflexes, and activities of the central nervous system (posture, exploratory movements, stereotypes, presence of clonic, or tonic movements) and the autonomic nervous system (respiratory pattern, piloerection, and pupil size).

### 2.5. Induction of Experimental Diabetes

The induction of diabetes was performed using a single injection of 150 mg/Kg streptozotocin (STZ—Sigma—St. Louis, MO) in mice which fasted for 12 to 14 hours. The STZ was dissolved in citrate buffer (pH 4.5) and immediately injected intraperitoneally. The animals kept fasting for 3 hours after injection. In the next 24 hours the animals received a glucose solution of 3% to drink to prevent hypoglycemia. After a period of seven days, the mice fasted again (10 to 12 hours) and blood glucose was measured with glucometer (One Touch, Johnson & Johnson). The animals with fasting glucose higher than 250 mg/dL were considered diabetic and were included in the study.

### 2.6. Treatment

The mice were randomly divided into 4 groups (*n* = 6–10/group) of control and diabetic animals (CTL SAL—nondiabetic mice treated with saline, CTL EXT—nondiabetic mice treated with extract, STZ SAL—diabetic mice treated with saline, and STZ EXT—diabetic mice treated with extract). Saline (1 mL/Kg b.w.) and extract of the leaves of* Myrcia bella* (300 and 600 mg/Kg b.w., dissolved in saline) were administered orally by gavage once a day during 14 consecutive days.

### 2.7. Fasting Blood Glucose, Body Weight, and Food and Water Intake

The fasting blood glucose was measured weekly using a glucometer (One Touch, Johnson & Johnson). Body weight and water and food intake were monitored daily.

### 2.8. Intraperitoneal Glucose Tolerance Test (ipGTT)

The animals from the 4 groups (*n* = 8) of control and diabetic mice treated during 14 days with saline or extract of* Myrcia bella* (600 mg/Kg) fasted (10–12 h) and the fasting blood glucose was measured before the glucose load and defined as time 0. A glucose load (2 g/kg b.w.) was injected intraperitoneally after 30 minutes of extract or saline administrations. Blood glucose was collected from tail tip 15, 30, 60, and 90 minutes after the administration of the glucose and measured by commercial kits of enzymatic glucose (Doles, Goiás, Brasil).

### 2.9. Biochemical Parameters

At the end of treatment period, the animals were killed and blood samples were collected and centrifuged to obtain the serum. Total proteins, total cholesterol, and triglycerides were measured by commercial kits (Doles, Goiás, Brazil). Basal plasma insulin was measured using a rat/mouse insulin ELISA kit (Millipore, St. Charles, MO) in spectrophotometer (BioTek-PowerWave XS) according to the manufacturers.

### 2.10. Hepatic and Muscular Glycogen

Hepatic and muscular glycogen content was measured as previously described by Rafacho et al. [17]. Briefly, liver and muscle samples (300 to 500 mg) were transferred to test tubes containing 30% KOH (Mallinckrodt Baker, Paris, France) and boiled for 1 h until they were completely homogenised. Na_2_SO_4_ (Mallinckrodt Baker, Paris, France) was then added, and glycogen was precipitated with 100% ethanol. The samples were centrifuged at 800 g for 10 min, the supernatants were discarded, and the glycogen was dissolved in hot distilled water. Ethanol was added, and pellets were obtained after a second centrifugation. The pellets were then dissolved in distilled water to a final volume of 20 mL. The glycogen content was measured by treating a fixed volume of samples with phenol reagent and H_2_SO_4_ and the absorbance was read at 490 nm with a spectrophotometer (BioTek-PowerWave XS).

### 2.11. Protein Expression by Western Blotting

Livers from animals of all groups were collected and homogenized in an ice cold cell lyses buffer (Cell Signaling, Beverly, MA). Protein concentration was measured using the Bradford method, according to the manufacturer (Bio-Rad, Hercules, CA). Aliquots of total protein (100 *μ*g) were boiled at 100°C for 4 minutes in 30% of the volume in Laemmli buffer. The samples were fractionated on electrophoresis system (Mini Protean II, Bio-Rad, Hercules, CA) in polyacrylamide gel with the appropriate pore size for each molecular weight. After electrophoresis, the proteins were transferred to a nitrocellulose membrane (Bio-Rad, Hercules, CA) in the presence of 20% methanol and 0.02% SDS at constant voltage of 120 Volts. The membrane was then saturated with blocking solution containing 5% BSA basal solution (10 mM Trisma base, 150 mM NaCl, 0.05% Tween 20) overnight. Afterwards, the membrane was washed with basal solution at room temperature (RT). The proteins were detected in the membrane after incubation for 3 h at 4°C with primary antibody (Santa Cruz Biotechnology, CA, USA) specific for each protein in basal solution containing 3% albumin, followed by washing and incubation with peroxidase conjugated secondary antibody-HRP solution containing basal solution 1% skimmed milk at RT. The membranes were washed and incubated in dark room with luminol chemiluminescent substrate (Pierce, Rockford, IL) and exposed to autoradiographic film (Kodak T-Mat G/RA, Rochester, NY). The intensities of the bands were quantified by densitometry (Epson Expression 1600, Long Beach, CA).

### 2.12. Statistical Analyses

The results were expressed as Means ± SEM. For multiple comparisons ANOVA was used followed by* Tukey's posttest* and for comparison of two groups, Student's *t*-test was used. The level of significance adopted was *P* < 0.05.

## 3. Results

### 3.1. Chemical Profiling and Identification

**Figure 1:**
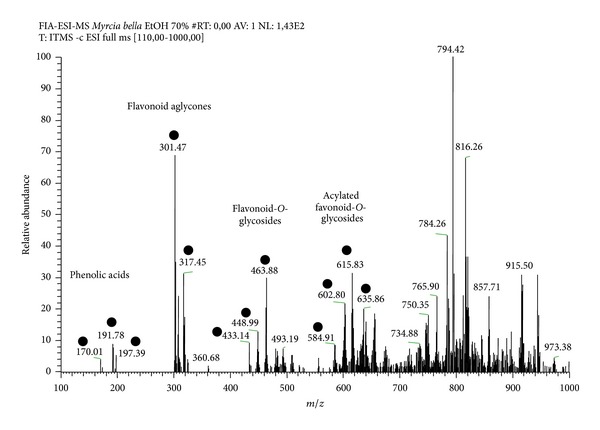
Chemical profile of* Myrcia bella's* extract. Typical direct flow injection analysis FIA-ESI-IT-MS fingerprint spectra obtained in negative ion mode of the 70% EtOH from the leaves of* M. bella*. (●) Characteristics constituents fragmented.

The FIA-ESI-MS/MS^*n*^ analysis of the 70% EtOH extract highlighted the presence of precursor ions related to phenolic acids, flavonoid aglycones, flavonoid-*O*-glycosides, and acylated flavonoid-*O*-glycosides (Figure 1). The precursor ions at* m/z* 169 [M–H]^−^, 191 [M–H]^−^, and 197 [M–H]^−^ confirm the presence of phenolic acids. Second-generation product ion spectra of precursor ions at* m/z* 433 [M–H]^−^,* m/z* 447 [M–H]^−^,* m/z* 463 [M–H]^−^,* m/z* 585 [M–H]^−^,* m/z* 601 [M–H]^−^,* m/z* 615 [M–H]^−^, and* m/z* 635 [M–H]^−^ confirm the presence of flavonoid-*O*-glycosides and acylated flavonoid-*O*-glycosides. The neutral losses of 132, 162, 146, and 152 mass units allowed the identification of pentosides (xylose or arabinose), hexosides (glucose or galactose), deoxyhexoside (rhamnose), and gallic acid, respectively. The precursor ions at* m/z* 301 and 317 characterized aglycones as quercetin and myricetin, respectively.

### 3.2. Toxicity Test

**Figure 2:**
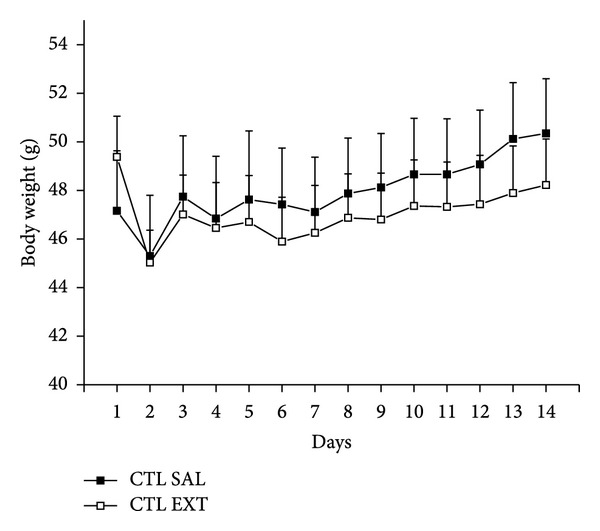
Body weight during acute toxicity test on male normoglycemic mice. Effect of acute administration of saline (CTL SAL) or crude extract of* M. bella* (2,000 mg/Kg-CTL EXT) on the body weight of normoglycemic mice. Data are Means ± SEM, *n* = 10, *P* < 0.05.* Student's t-test.*

The extract of* Myrcia bella* administrated to normal mice at a dose of 2,000 mg/Kg did not provoke deaths nor changes in the behavioral parameters analyzed (posture, secretions, and presence of convulsions) when compared with the group who received saline. There was no difference in body weight of the animals in both groups (Figure 2). The kidney weights from CTL EXT animals increased significantly (14.27 ± 0.47 g/Kg) when compared with CTL SAL animals (12.73 ± 0.41 g/Kg) (Table 1, *P* < 0.05).

### 3.3. Effect of the Treatment with the Extract of* Myrcia bella *on Fasting Blood Glucose

**Figure 3:**
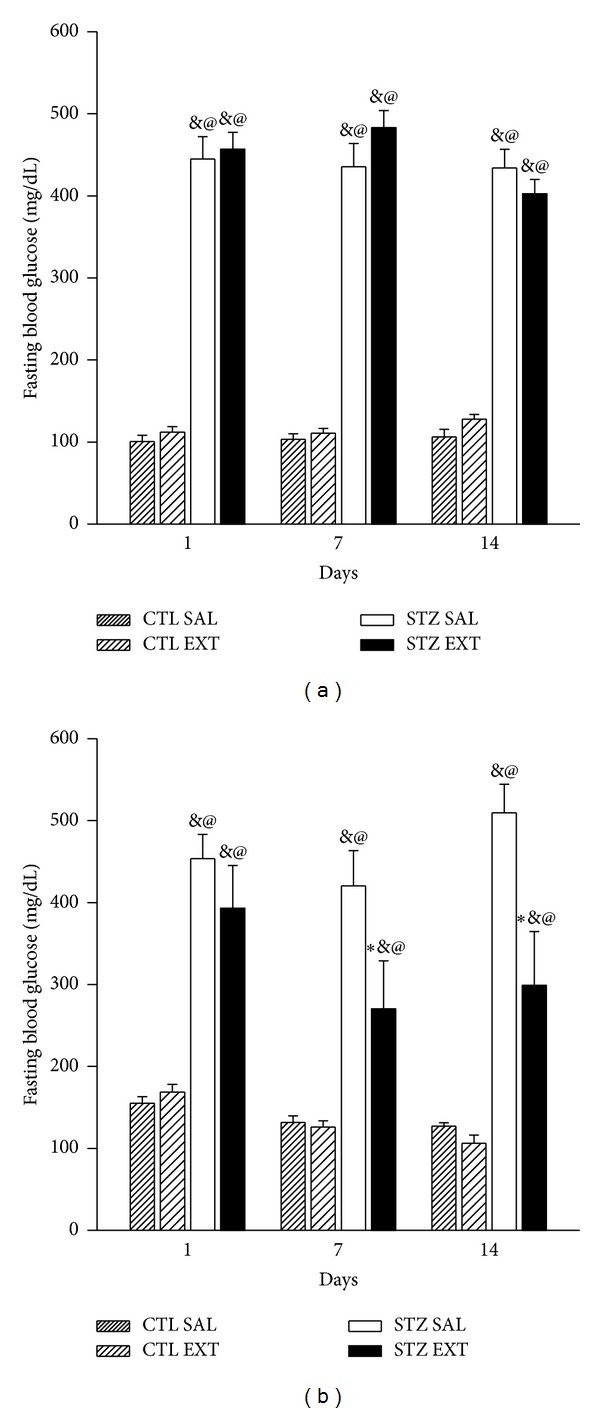
Effect of* Myrcia bella* administration (300 and 600 mg/Kg) on fast blood glucose. Fast blood glucose (mg/dL) of control and diabetic mice treated with saline (CTL SAL and STZ SAL), 300 mg/Kg (a) or 600 mg/Kg (b) of the crude extract of* M. bella *(CTL EXT and STZ EXT) during 14 days. Data are Means ± SEM. ∗ versus STZ SAL; @ versus CTL SAL, & versus CTL EXT, *n* = 10, *P* < 0.05. ANOVA followed by* Tukey's posttest.*

Diabetic mice (STZ SAL and STZ EXT) showed a significant increase in fasting blood glucose when compared with control groups (CTL SAL and CTL EXT) (*P* < 0.001). Diabetic mice treated with the extract of* M. bella* at 300 mg/Kg (STZ EXT) showed no decrease in fasting blood glucose when compared with diabetic animals treated with saline (STZ SAL) (Figure 3(a)). However, STZ EXT animals treated with 600 mg/Kg of the extract of* M. bella* (STZ EXT) showed a significant decrease in fasting blood glucose from the 7th day to the last day of treatment compared with STZ SAL; the decrease was maintained until the end of the treatment (Figure 3(b)) (*P* < 0.05). On the 7th day of treatment, there was a 36% decrease in fasting glucose of STZ EXT animals compared with animals of the STZ SAL group. On the 14th day, the decrease reached 41.3% between the two groups. There was no difference in fasting blood glucose between the control groups (CTL SAL and CTL EXT). Because the treatment with the crude extract of* Myrcia bella* was effective in lowering blood glucose at a dose of 600 mg/Kg, the dose was used for the rest of the experiments.

### 3.4. Effect of the Treatment with the Extract of* Myrcia bella *on ipGTT

**Figure 4:**
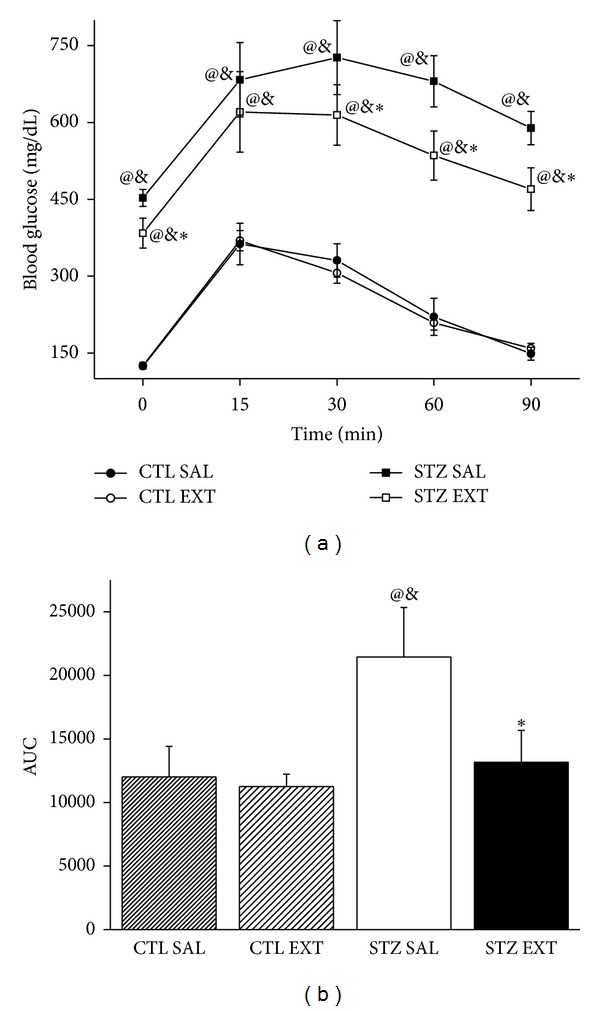
Intraperitoneal glucose tolerance test. Glycemia after intraperitoneal glucose administration (2 g/Kg) (a) and area under the curve (AUC) (b) in control and diabetic mice treated with saline (CTL SAL and STZ SAL) or with 600 mg/Kg of the crude extract of* M. bella *(CTL EXT and STZ EXT) during 14 days (CTL EXT and STZ EXT). Data are Means ± SEM. ∗ versus STZ SAL; @ versus CTL SAL, & versus CTL EXT, *n* = 8, *P* < 0.05. ANOVA followed by* Tukey's posttest.*

The ipGTT was performed according to the effective hypoglycemic dose of the extract (600 mg/Kg). Diabetic animals treated with the extract (STZ EXT) showed a significant decrease in glycemia at 30, 60, and 90 minutes (*P* < 0.05) after glucose administration (2 g/Kg) compared with diabetic animals treated with saline (STZ SAL) (Figure 4(a)). The area under the curve (AUC) (Figure 4(b)) was increased in both diabetic groups when compared with the control groups (CTL SAL and CTL EXT) (*P* < 0.001). Nevertheless, there was a decrease in AUC of the STZ EXT group when compared with the STZ SAL group (*P* < 0.05). There was no difference in blood glucose and AUC between the control groups.

### 3.5. Effect of the Treatment with the Extract of* Myrcia bella *on Body Weight and Food and Water Intake

**Figure 5:**
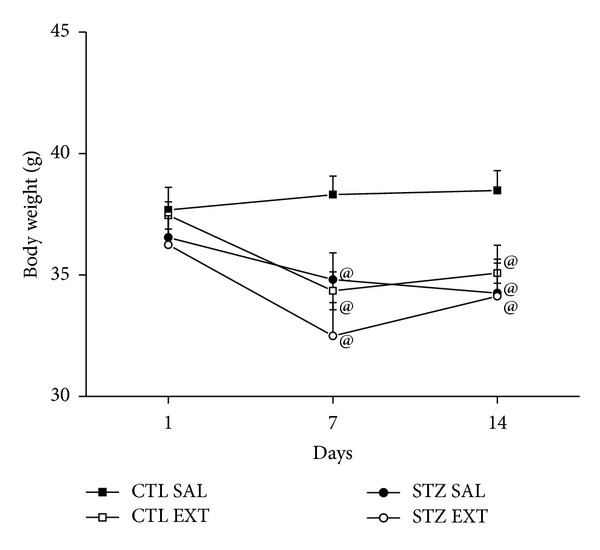
Effect of* Myrcia bella* administration (600 mg/Kg) on the body weight. Body weight (g) of control and diabetic animals treated with saline (CTL SAL and STZ SAL) or with 600 mg/Kg of the crude extract of* M. bella *during 14 days (CTL EXT and STZ EXT). Data are Means ± SEM. @ versus CTL SAL, *n* = 8, *P* < 0.05. ANOVA followed by* Tukey's posttest.*

**Figure 6:**
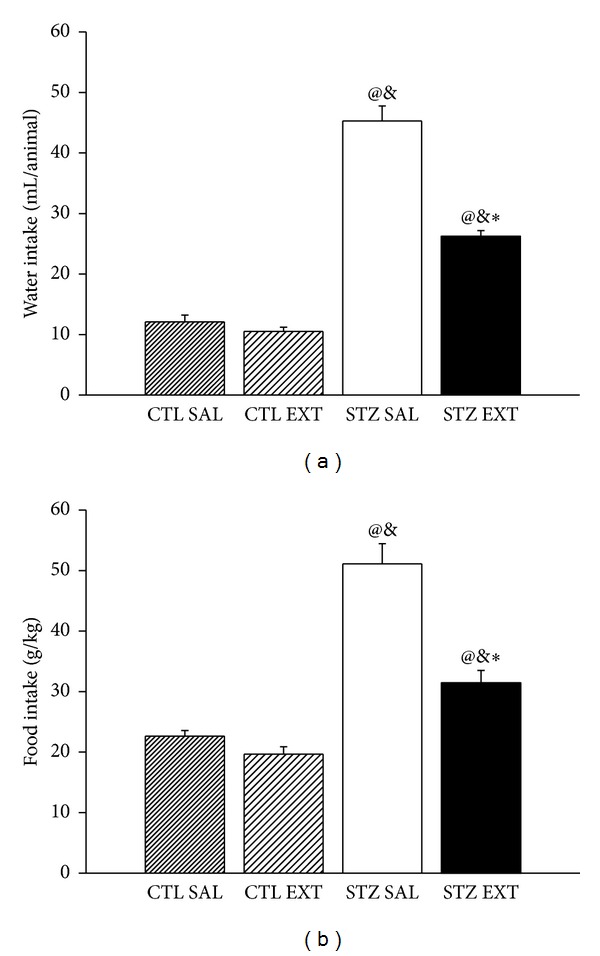
Effect of* Myrcia bella* administration (600 mg/Kg) on water and food intake. Water intake (mL/animal) (A) and food intake (B) of control and diabetic mice treated with saline (CTL SAL and STZ SAL) or with 600 mg/Kg of the crude extract of* M. bella* (CTL EXT and STZ EXT) during 14 days. Data are Means ± SEM. ∗ versus STZ SAL; @ versus CTL SAL, & versus CTL EXT, *n* = 8, *P* < 0.05. ANOVA followed by* Tukey's posttest.*

The body weight of the diabetic animals (STZ SAL and STZ EXT) showed a significant decrease compared with the body weight of the control group treated with saline (CTL SAL) as of the 7th day of treatment (Figure 5) (*P* < 0.05). However, there was no significant difference between body weights of diabetic animals of the groups STZ SAL and STZ EXT. There was a decrease of body weight of control animals treated with the extract (CTL EXT) compared with control animals treated with saline (CTL SAL) as of the 7th day of treatment (*P* < 0.05). Diabetic animals (STZ SAL and STZ EXT) showed a significant increase in water intake compared to the control groups (CTL SAL and CTL EXT) (Figure 6(a)) (*P* < 0.001). Diabetic animals treated with the extract of* M. bella* (STZ EXT) showed a decrease of 42% in water intake when compared to diabetic mice treated with saline (STZ SAL) (*P* < 0.05). There was no difference between the control groups (CTL SAL and CTL EXT). Regarding food intake, diabetic animals (SAL STZ and STZ EXT) showed a significant increase compared with the control groups (CTL SAL and CTL EXT) (Figure 6(b)) (*P* < 0.001). Diabetic animals treated with a crude extract of* M. bella* (STZ EXT) showed a decrease of 38.46% in food intake compared with diabetic animals treated with saline (STZ SAL) (*P* < 0.001). There was no difference between the control groups (CTL SAL and CTL EXT).

### 3.6. Biochemical Parameters

Total triglycerides (TG) and cholesterol (TC) were increased in diabetic animals treated with saline (STZ SAL) compared with control groups (CTL SAL and CTL EXT) (*P* < 0.05). The treatment with the extract of* Myrcia bella* at 600 mg/Kg for 14 days decreased the TG and TC levels of the animals from STZ EXT and CTL EXT groups when compared with the animals of the STZ SAL and CTL SAL groups, respectively (*P* < 0.05). The decrease was 49.65% and 45.5% in the levels of TG of STZ EXT and CTL EXT compared to STZ SAL and CTL SAL, respectively, and 53.4% and 48.6% in the levels of TC of STZ EXT and CTL EXT compared to STZ SAL and CTL SAL, respectively. There was no difference in total blood proteins between the groups. Basal plasma insulin decreased in STZ SAL (0.11 ± 0.005 ng/mL) and STZ EXT (0.13 ± 0.01 ng/mL) when compared with CTL SAL (0.19 ± 0.02 ng/mL) and CTL EXT (0.19 ± 0.01 ng/mL) (*P* < 0.05). There was no difference between basal insulin of STZ SAL and STZ EXT (Table 2).

### 3.7. Hepatic and Muscular Glycogen

The hepatic and muscular glycogen contents in diabetic mice treated with saline (STZ SAL) decreased compared to the control groups (CTL SAL and CTL EXT) (*P* < 0.05). The treatment with* Myrcia bella *increased hepatic glycogen content in diabetic mice (0.28 ± 0.06 mg%) when compared with the diabetic animals treated with saline (0.11 ± 0.03 mg%) (*P* < 0.05). There was no difference between control groups (CTL SAL and CTL EXT). There was no difference in the muscular glycogen content of diabetic STZ EXT and STZ SAL and between control groups (Table 2).

### 3.8. Protein Expression by Western Blotting

**Figure 7:**
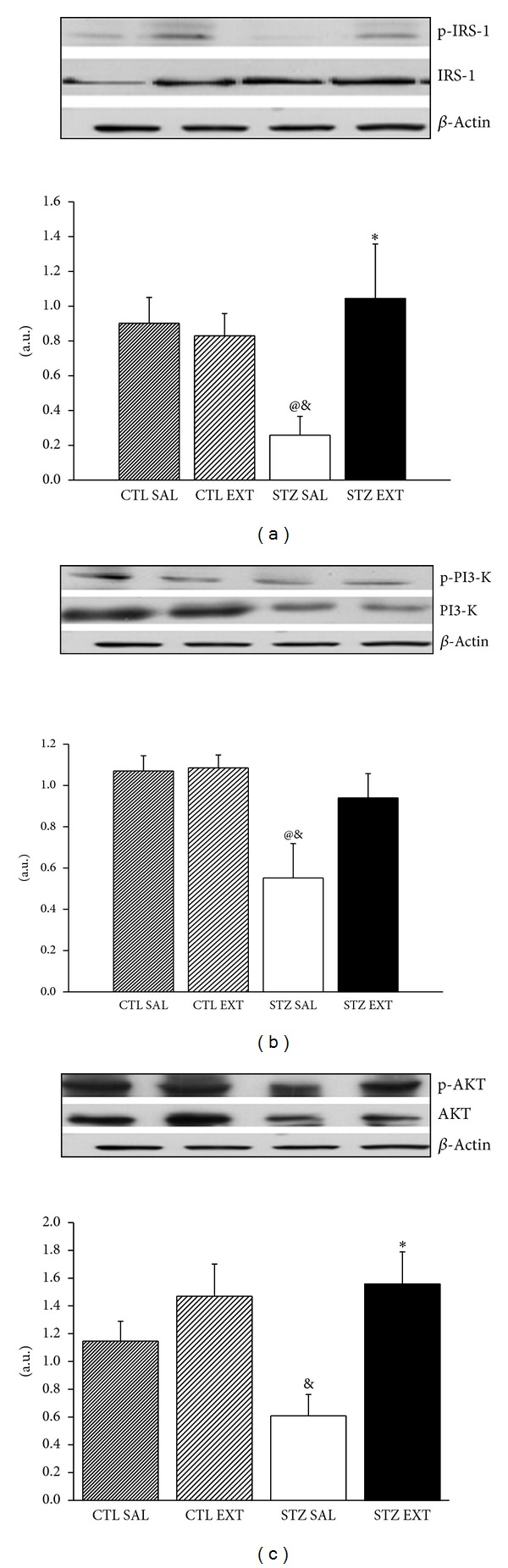
Protein expression in liver. Western blot analysis of IRS-1 (a), PI3-K(b), and AKT (c) from liver of control and diabetic mice treated with saline (CTL SAL and STZ SAL) or with 600 mg/Kg of the crude extract of* M. bella* during 14 days (CTL EXT and STZ EXT). p-IRS-1, p-PI3-K, and P-AKT are the phosphorylated forms of the respective proteins. Data are Means ± SEM. ∗ versus STZ SAL, @ versus CTL SAL, & versus CTL EXT, *n* = 6, *P* < 0.05. ANOVA followed by* Tukey's posttest.*

The expression of total and phosphorylated levels of IRS-1, PI3-K, and AKT in the livers of the animals of all groups was measured. The relative expression of p-IRS-1/IRS-1 (Figure 7(a)), p-PI3-K/PI3-K (Figure 7(b)), and p-AKT/AKT (Figure 7(c)) showed a significant increase in STZ EXT (1.04 ± 0.3; 0.95 ± 0.16; 1.5 ± 0.23, resp.) when compared with animals of the STZ SAL group (0.25 ± 0.1; 0.55 ± 0.1; 0.88 ± 0.07, resp.) (*P* < 0.05). There was no difference between the control groups.

## 4. Discussion

In this study, we investigated the hypoglycemic activity of the extract of leaves of* Myrcia bella* administered to streptozotocin-induced diabetic mice. The selected dose for the treatment of animals was chosen according to the effectiveness of the tested doses. The treatment with the crude extract of* Myrica bella *using 300 mg/Kg b.w. for 14 days did not reduce the hyperglycemia of the diabetic animals. Once the treatment with the extract of* Myrcia bella* was effective in lowering blood glucose at the dose of 600 mg/Kg for 14 days, this dose was chosen to carry out the proposed experiments.

The popular use of species of* Myrcia* is well known.* Myrcia multiflora*, known as “pedra-hume-de caá,” is used in folk medicine for the treatment of diabetes, diarrhea, enteritis, and hemorrhages [10, 11, 13].* Myrcia bella *is a species belonging to the Myrtaceae family that occurs in the Brazilian cerrado, and its hypoglycemic effects have not previously been investigated.

Hyperglycemia resulting from DM is a consequence of defects or a lack of insulin secretion and/or action [1]. The studies with experimental models of diabetes in rodents have been widely used with the intent to reproduce the symptoms of diabetes. The most common substances used to induce diabetes in rodents are alloxan and streptozotocin [18]. The animals induced to diabetes by STZ show symptoms such as polyphagia, polydipsia, polyuria, and weight loss, as seen in our streptozotocin-injected animals. Water and food intake decreased in animals of the STZ EXT group when compared with the animals of the STZ SAL group. Pepato et al. [19] showed that the extract of* Myrcia uniflora* decreased polyphagia and polydipsia and reduced the hyperglycemia in streptozotocin-induced diabetic animals. Our present results are in agreement with this report. Body weight was decreased in diabetic animals, and the treatment with the extract did not alter weight loss. An interesting finding was the effect of the extract in the control mice. There was a significant decrease in the weight of those animals, as well as in triglycerides and total cholesterol. Since weight loss is desirable in many situations, particularly in preventing the development of type 2 diabetes, this effect should be investigated further. According to Table 1 and to the behavioral analysis, the extract seems to be nontoxic to the animals, although the ratio of kidney weight to body weight had an increase of 12.1% with the administration of high dose of the extract (2,000 mg/kg), indicating a possible degree of toxicity of the extract when in excess. However, the urinary rate was not affected in this situation.

After 14 days of treatment with the extract of* M. bella* at 600 mg/Kg, we observed a significant reduction of the hyperglycemia in the treated diabetic mice (STZ EXT) when compared with diabetic mice treated with saline (STZ SAL). The reduction began at the 7th day of the treatment and remained until the end, proving the hypoglycemic effect of the treatment. The ipGTT showed a significant increase in glucose tolerance of STZ EXT animals when compared with STZ SAL animals, which indicates a better handling of glucose.

The chemical fingerprinting of the 70% EtOH leaves extract by FIA-ESI-IT-MS confirmed the presence of the main characteristic constituents of* Myrcia bella* as phenolic acids and flavonoids [15]. Flavonoids present a variety of biochemical and pharmacological actions that may affect the function of various mammalian cell systems [20]. Several studies have shown that flavonoids, which are naturally occurring phenolic compounds that are widely distributed in plants, can act in lowering blood glucose in experimental models of diabetes. Such studies using isolated flavonoids, such as quercetin and myricetin, showed a reduction in the fasting blood glucose of diabetic rats induced by streptozotocin [21–23]. Vassal et al. [22] showed that isolated quercetin administered at 10 and 15 mg in streptozotocin-induced diabetic rats reduced fasting blood glucose, total cholesterol, and triglycerides as it regenerates the pancreatic islet. Ong and Khoo [21] found that myricetin administered for 2 days at 3 mg/12 h reduced glycemia in 50% of diabetic rats as hepatic glycogen increased, indicating that myricetin should have an effect on glycogen metabolism. Accordingly, the flavonoids present in the crude extract obtained from leaves of* M. bella* should assist in the reduction of blood glucose through mechanisms that involve the reduction of hepatic glucose production and/or that promote the storage of glucose in the liver. The presence of phenolic acids was also detected in the extract of* M. bella*. This group of substances is characterized by their antioxidant properties. Also, it was demonstrated that caffeic acid and chlorogenic acid increased glucose uptake in L6 muscular cells and stimulate insulin secretion from the INS-1E insulin-secreting cell line and rat islets of Langerhans [24]. Phenolic acids also have the capacity to inhibit enzymes like *α*-glucosidase [25] and protein tyrosine phosphatase 1B (PTP1B) [26]. In this study, the increase in p-IRS-1 expression, discussed below, arises the possibility for the participation of phenolic acids in the observed effects of the extract of* M. bella*.

Glycogen is the form in which some tissues store glucose. Insulin stimulates the accumulation of glycogen by increasing glucose transport in muscle and glycogen synthesis in the liver and in muscle [27]. In diseases such as diabetes, hepatic and muscular glycogen is decreased, as seen in diabetic mice treated with saline in this study (STZ SAL). The treatment with the extract of* M. bella* augmented the content of the glycogen in the liver of STZ EXT animals when compared with STZ SAL. This increase may occur through the activation of glycogenesis and/or inhibition of processes such as glycogenolysis and gluconeogenesis, all of which are regulated by insulin [27]. The glycogen content in the muscles of diabetic animal treated with the extract was not affected by the treatments, suggesting that muscle tissue is not significantly involved in glucose handling in this situation.

Dyslipidemia is frequently found among the complications in diabetic patients and represents a serious risk for cardiovascular disease. The most common lipid abnormalities found in patients with diabetes are hypertriglyceridemia and hypercholesterolemia [28]. The levels of total cholesterol and triglycerides increased in the diabetic animals of the STZ SAL group. The treatment with the extract of* M. bella* reduced the levels of TC and TG in animals in the STZ EXT and CTL EXT groups when compared with STZ SAL and CTL SAL, respectively. These results indicate a possible beneficial hypolipidemic activity of* M. bella* extract. The intake of flavonoids and other phenolic compounds is associated with a decreased risk of cardiovascular diseases, since these compounds are involved in lowering lipid levels and decreasing LDL oxidation [20]. Among the multiple effects of flavonoids, they have antioxidant properties as free radical scavengers by chelating metal ions, thereby protecting cells from free radicals and therefore lipid peroxidation [29]. Flavonoids may also reduce the oxidation of excess lipids in blood circulation by ROS that can generate cardiovascular problems [30, 31]. It can be suggested, therefore, that the presence of flavonoids in the extract administered to the mice may have affected lipid metabolism by lowering the levels of triglycerides and cholesterol.

The extract of* Myrcia bella* did not alter the plasma insulin levels of treated diabetic mice. These results indicate a possible extrapancreatic action of the extract, as there was no difference in basal insulin levels between diabetic mice treated with saline and the extract.

In order to further investigate the mechanisms involved in the reduction of blood glucose levels of diabetic animals and in the increase in storage of glucose in the liver, the expressions of some key proteins at the insulin signaling pathway were analyzed. The PI3-K/AKT pathway is the primary pathway activated by insulin through its receptor (IR). Upon its activation, a cascade of events mediated by protein-protein interactions takes place, which leads to the metabolic actions of insulin. The PI3-K activated by insulin is responsible, among other functions, for the phosphorylation and activation of AKT, which, in phosphorylated form, is responsible for both storing and preventing the degradation of glucose in the liver [27]. The treatment with* M. bella* in diabetic animals (STZ EXT) leads to an increase in the expressions of IRS-1, PI3-K, and AKT in comparison with the diabetic animals treated with saline (SAL STZ), indicating that the hypoglycemic action of the extract, at least in part, involves the activation of the insulin signaling pathway involving PI3-K/AKT proteins. The molecular mechanisms by which flavonoids and phenolic acids stimulate the expression of proteins are unknown, but this is a current found effect described in the literature.

The data showed in this study indicates that the improvement of glucose metabolism in STZ EXT should occur through an increase in the expression of key proteins of the insulin pathway that activate the process of glycogenesis and inhibit the processes of gluconeogenesis and glycogenolysis. Moreover, the extract can act as a hypolipidemic agent, given the decreased levels of serum lipids. The characterization of the extract of* Myrcia bella* as a hipoglicemic and hypolipidemic agent opens an interesting field of investigation, requiring further studies about the mechanisms involved.

## Figures and Tables

**Table 1:** Effect of the acute administration of saline (1 mL/Kg) or crude extract of *M. bella* (2000 mg/Kg) on organs weight of male mice.

Organs weight (g/Kg)	CTL SAL	CTL EXT
Spleen	4.04 ± 0.32	4.71 ± 0.53
Heart	4.81 ± 0.12	5.06 ± 0.24
Liver	55.85 ± 1.72	58.66 ± 1.29
Lung	8.94 ± 0.92	9.67 ± 0.36
Kidney	12.73 ± 0.41	14.27 ± 0.47*

CTL SAL (control mice treated with saline), CTL EXT (control mice treated with *M. bella *extract), STZ SAL (diabetic mice treated with saline), and STZ EXT (diabetic mice treated with *M. bella *extract). Values are expressed as Means ± SEM. * versus CTL SAL, *n* = 10, *P* < 0.05.

**Table 2:** Biochemical parameters and glycogen levels of control and diabetic mice treated during 14 days with saline or crude extract of *M. bella* (600 mg/Kg).

Parameters	CTL SAL	CTL EXT	STZ SAL	STZ EXT
Triglycerides (mg/dL)	105.1 ± 12.5	79 ± 17.88^@^	139.4 ± 12.6^@&^	70.2 ± 8.18^@∗^
Total cholesterol (mg/dL)	142.9 ± 12	83.6 ± 14.8^@^	190 ± 13.8^@&^	88.5 ± 11.3^@∗^
Total protein (mg/dL)	4.89 ± 1.37	3.9 ± 0.94	2.7 ± 0.47	3.59 ± 0.37
Basal insulin (ng/mL)	0.19 ± 0.02	0.19 ± 0.01	0.11 ± 0.005^@&^	0.13 ± 0.01^@&^
Hepatic glycogen (mg%)	0.33 ± 0.07	0.33 ± 0.05	0.11 ± 0.03^@&^	0.28 ± 0.06*
Muscular glycogen (mg%)	0.21 ± 0.01	0.21 ± 0.02	0.15 ± 0.01^@&^	0.17 ± 0.01^@&^

CTL SAL (control mice treated with saline), CTL EXT (control mice treated with *M. bella *extract), STZ SAL (diabetic mice treated with saline), and STZ EXT (diabetic mice treated with *M. bella *extract). Values are expressed as Means ± SEM. * versus STZ SAL, ^@^ versus CTL SAL, ^&^ versus CTL EXT, *n* = 8, *P* < 0.05. ANOVA followed by *Tukey's posttest*.
